# In Vivo Engraftment and Functional Efficacy of a 3D-Bioprinted Human Parathyroid Equivalent

**DOI:** 10.3390/medicina62030442

**Published:** 2026-02-26

**Authors:** Sumeyra Guler, Seyda Gokyer, Suleyman Can Oztürk, Ertugrul Çelik, Hamdullah Yanik, Ibrahim Burak Bahcecioglu, Mehmet Ali Gulcelik, Pinar Yilgor, Kerim Bora Yilmaz

**Affiliations:** 1 Department of Surgical Oncology, Ankara Gulhane Training and Research Hospital, University of Health Sciences, Ankara 06010, Turkey; guler.sumeyra@yahoo.com (S.G.); dr.ibb@hotmail.com (I.B.B.); mgulcelik@yahoo.com (M.A.G.); 2 Department of Biomedical Engineering, Ankara University, Ankara 06100, Turkeyphuri@ankara.edu.tr (P.Y.); 3 Research and Application Center for Animal Experiments, Hacettepe University Cancer Institute, Ankara 06100, Turkey; 4 Department of Pathology, Ankara Gulhane Training and Research Hospital, University of Health Sciences, Ankara 06010, Turkey; 5 Department of Basic Oncology, Hacettepe University Cancer Institute, Ankara 06100, Turkey; yanikhamdullah@gmail.com; 6 Ankara University Medical Design Research and Application Center (MEDITAM), Ankara 06520, Turkey; 7 Department of Medical and Surgical Research, Institute of Health Sciences, Hacettepe University, Ankara 06800, Turkey; 8 Department of General Surgery, Gulhane Training and Research Hospital, University of Health Sciences, Ankara 06010, Turkey

**Keywords:** parathyroid, 3D bioprinting, surgical hipoparathyroidism, hypocalcemia, in vivo model

## Abstract

*Background and Objectives*: Hypocalcemia due to hypoparathyroidism (HypoPTH) is the most common complication following thyroid surgery, typically resulting from iatrogenic removal, tissue damage, or compromised vascularization of the parathyroid glands. Patients with persistent HypoPTH are at risk for long-term complications such as osteoporosis, cardiac dysfunction, and renal impairment. Lifelong regulation of calcium levels is therefore essential to prevent morbidity and mortality associated with these complications. In this study, we aimed to evaluate the functional engraftment efficacy of 3D bioprinted human parathyroid tissue constructs in a xenograft model in vivo. *Materials and Methods*: Primary cells obtained from freshly excised human parathyroid tissue specimens were isolated and 3D bioprinted using alginate-based bioink. The bioprinted tissue constructs were implanted into CD1 athymic mice. Histopathological evaluation of the grafted constructs was performed at different time points. In addition, surface calcium-sensing receptor (CaSR) expression was assessed by immunofluorescence as an indicator of functional parathyroid tissue engraftment. *Results*: The presence of CaSR on parathyroid cells within the 3D-printed scaffolds confirmed the persistence of functional parathyroid cells following implantation. In tissue samples obtained during the first, second, and third weeks after implantation, CaSR positivity was consistently observed in the parathyroid cells. However, at the three-month follow-up, the pores within the scaffolds were found to be filled with calcified material and replaced by fibrotic tissue. At this stage, the absence of parathyroid hormone (PTH) expression indicated a loss of functional activity in the grafted biomaterial. *Conclusions*: Human primary parathyroid cells were successfully isolated, and a functional, hormone-active parathyroid tissue substitute was developed ex vivo using 3D-bioprinted hydrogel scaffolds combined with autologous cells. Although short-term functional engraftment was achieved, long-term graft viability and hormonal activity were limited due to scaffold degradation and fibrosis. These findings indicate the necessity for further improvement in scaffold biocompatibility to enhance the therapeutic potential of 3D-bioprinted parathyroid tissue constructs for in vivo applications.

## 1. Introduction

Hypocalcemia due to hypoparathyroidism (HypoPTH) is the most common complication following thyroid surgery. This condition arises from inadvertent removal or damage to the parathyroid glands or their vascularization during surgery [1,2]. It is more frequently observed in thyroid cancer patients undergoing central neck dissection, in patients with a background of thyroiditis where surgical dissection is more challenging, and in those with Graves’ disease undergoing surgery [3,4,5,6]. Both surgical technique and the extent of thyroidectomy are critical factors influencing parathyroid gland injury [7,8]. While transient postoperative HypoPTH, typically resolving within six months, is common, permanent hypocalcemia occurs less frequently. The incidence of transient HypoPTH ranges between 20% and 40% in different postoperative studies [9,10]. In contrast, permanent PHipo and the associated hypocalcemia occur at rates ranging from 1% to 15% [11,12].

Hypocalcemia typically manifests on the first day following surgery. HypoPTH leads to hypocalcemia due to impaired secretion of Parathyroid Hormone (PTH), resulting in an inability to mobilize calcium from bone, reabsorb calcium from the distal nephron, and stimulate 1α-hydroxylase activity in the kidney [1]. The hallmark features include hypocalcemia, elevated serum phosphorus levels, and low plasma PTH levels. Clinically, HypoPTH becomes symptomatic in the early postoperative period, often presenting with paresthesia, weakness, muscle cramps, and tetany due to increased neuromuscular excitability. In rare cases, life-threatening complications such as seizures, laryngospasm, bronchospasm, and cardiac arrhythmias may occur [1,13]. While transient HypoPTH generally resolves, predicting which patients will develop permanent hypoparathyroidism is challenging. Those with significantly low early postoperative PTH levels and severe symptoms are at greater risk for permanent HypoPTH and may require lifelong treatment for hypocalcemia. Patients with permanent HypoPTH face long-term risks including osteoporosis, cardiac and renal dysfunction, and psychological challenges. It is crucial to maintain regulated calcium levels throughout their lives to prevent complications that could lead to morbidity and mortality. Patients with persistent HypoPTH experience a diminished quality of life, and long-term HypoPTH has been linked to an increased risk of mortality [12,14].

The primary goal of medical management in HypoPTH is to achieve and maintain normocalcemia and normophosphatemia while ensuring the patient remains symptom-free. Treatment typically involves the administration of vitamin D or its analogs, alongside calcium supplements; however, this regimen does not fully compensate for the absence of PTH. Lifelong dose adjustments often necessitate frequent hospital visits. Given that many thyroid surgery patients are young, they face a lifetime of costly replacement therapy. Furthermore, these treatments carry the risk of inducing hypercalcemia and/or hypercalciuria, potentially leading to complications such as ectopic mineralization and renal failure [12]. The disruption of calcium regulation due to the loss of PTH and calcium homeostasis results in lifelong imbalances in bone, renal, and systemic calcium and phosphorus metabolism [12].

The tissue engineering approach enables the development of functional tissues under laboratory conditions. Three-dimensional (3D) bioprinting, a primary strategy within tissue engineering, holds significant potential for advancing personalized medicine [15]. This promise has spurred the development of biocompatible systems for 3D bioprinting, making it an exciting prospect for tissue engineering applications [16]. 3D bioprinters can create functional biological structures that may restore, maintain, enhance, or even replace organ functions [17]. By enabling the precise layer-by-layer positioning of living cells, bioprinting facilitates the creation of complex biological structures such as tissues and organs, paving the way for innovative transplantation research that is increasingly being translated into clinical practice [18,19,20].

Endocrine tissues are promising targets for 3D bioprinting due to their small size, relatively simple functional and anatomical structures, and the critical role their secretions play in maintaining hormonal homeostasis [21]. Although research on 3D bioprinting of endocrine organs remains limited, their straightforward structure and small size present a distinct advantage, as these characteristics are sufficient for functionality compared to more complex organs like the liver and kidneys [22].

In our study, we aimed to establish an in vivo model on athymic CD1 mice to assess the functional engraftment efficacy of 3D bioprinted human parathyroid tissue constructs.

## 2. Materials and Methods

### 2.1. Cell Isolation and Culture

Human parathyroid tissue was obtained in accordance with the Clinical Research Ethics Committee approval (University of Health Sciences, Gülhane Non-Interventional Clinical Research Ethics Committee, Approval No: 2024-560, Date: 10 December 2024) from patients who have undergone thyroidectomy. Patients who underwent unilateral thyroid surgery for benign reasons were included in the study. In case of intraoperative iatrogenic parathyroid devascularization of the patients, the tissue was autoimplanted between the sternocleidomastoid muscle fibers of the patient, and a maximum of 10% was separated for use in our experimental study.

During unilateral thyroidectomy at the University of Health Sciences, Ankara Gulhane Training and Research Hospital general surgery operating room (10:00 a.m.), parathyroid tissue that was intraoperatively identified as damaged was confirmed by frozen section histopathological analysis. Following confirmation, 75% of the tissue was autotransplanted, while the remaining 25% was transferred to the Biomedical Engineering Department at Ankara University. There, a parathyroid equivalent was created using a 3D printer after the isolation of human parathyroid cells (5:00 p.m.). These scaffolds were then implanted into CD1 athymic nude mice at Hacettepe University Cancer Institute Research and Application Center for Animal Experiments (7:00 p.m.). The entire process, which involved collaboration among three centers in different parts of Ankara, took a total of 9 h to produce and implant the parathyroid tissue replica using 3D modeling.

### 2.2. Cell Isolation and 3D Bioprinting

Cell isolation was performed as previously described [21]. Briefly, freshly excised tissue samples were first minced into small fragments (approximately 1–2 mm^3^) using a sterile sharp lancet under sterile conditions. The minced tissue sample was then transferred into a 50 mL conical tube containing 15 mL of enzymatic digestion solution, which consisted of 1.5 mL collagenase type II (Sigma Aldrich, St Louis, MO, USA) that digests collagen and 500 µL DNase I (Serva, Heidelberg, Germany) that removes the unwanted free genomic material, prepared in sterile 1x phosphate-buffered saline (PBS). The suspension was incubated at 37 °C for 1 h with gentle agitation on an orbital shaker to facilitate enzymatic dissociation of the extracellular matrix and to prevent DNA-mediated cell clumping.

Following digestion, the cell suspension was centrifuged at 3500 rpm for 10 min at room temperature to pellet the released cells. The supernatant was carefully discarded, and the pellet was resuspended in PBS. The resulting suspension was then passed through a 40 µm sterile cell strainer to remove undigested tissue fragments and obtain a single-cell suspension. The filtrate containing viable isolated cells was collected into a fresh sterile 50 mL falcon tube. Finally, the cells were counted, viability was checked by trypan blue exclusion, and the desired cell concentration was adjusted before transferring the suspension into the bioink for further applications.

**Figure 1:**
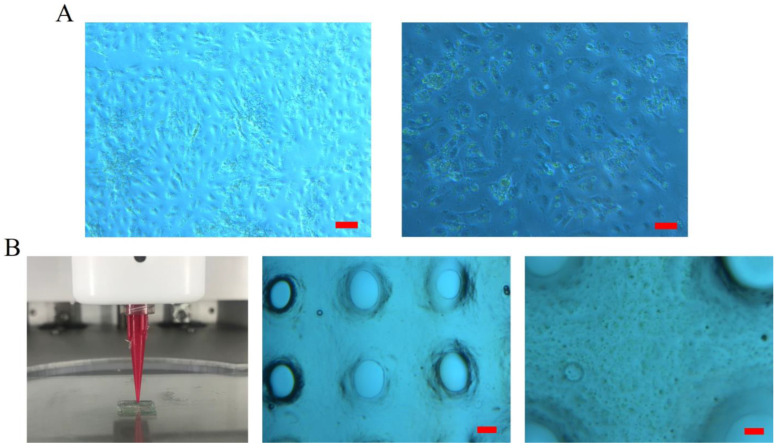
(**A**) Morphology of the isolated primary human parathyroid cells. (**B**) 3D bioprinting of the constructs, and cell distribution and morphology within the 3D scaffold.

Parathyroid cells were 3D bioprinted using the Axo A2 bioprinter system (Axolotl Biosystems, Ankara, Turkey) maintained at 37 °C under sterile conditions, as previously described [21] (Figure 1). Prior to bioprinting, alginate powder was sterilized by ultraviolet (UV) irradiation for 30 min to ensure the removal of potential contaminants. Under aseptic conditions, isolated parathyroid cells were homogeneously suspended in a 6% (*w*/*v*) aqueous alginate solution to prepare the cell-laden bioink. The final bioink was carefully loaded into sterile cartridges and bioprinted into predefined scaffold structures using the Axo A2 system. To ensure structural stability for in vivo applications, the bioprinted constructs were cross-linked using 0.5 M calcium chloride (CaCl_2_).

Immediately following fabrication, the freshly bioprinted constructs were gently collected and transplanted into the dorsal flanks of immunodeficient nude mice through a small subcutaneous incision. Implanted scaffolds were placed in direct contact with the host tissue to promote vascular ingrowth and nutrient exchange. In parallel, acellular scaffolds generated from the same 6% alginate solution without cells were fabricated and implanted as control constructs. All surgical and implantation procedures were carried out under sterile conditions and in accordance with institutional animal care guidelines.

3D bioprinted constructs were also maintained under in vitro culture conditions to evaluate their viability and functionality prior to implantation. Constructs were transferred into 6-well culture plates and immersed in RPMI-1640 medium (Gibco, Waltham, MA, USA) supplemented with 10% fetal bovine serum (FBS; Gibco) and 1% penicillin/streptomycin (Pen/Strep; Gibco). Cultures were incubated at 37 °C in a humidified atmosphere containing 5% CO_2_, and the medium was refreshed every 2–3 days to ensure optimal nutrient supply and waste removal.

### 2.3. Immunofluorescence

The expression of the parathyroid-specific marker, the calcium-sensing receptor (CaSR), was evaluated by immunofluorescence staining. Briefly, cell-laden 3D bioprinted scaffolds were fixed with 4% paraformaldehyde (PFA) for 15 min at room temperature and subsequently washed three times with phosphate-buffered saline (PBS) to remove residual fixative. Following fixation, samples were permeabilized with 0.1% Triton X-100 in PBS for 10 min and blocked with 0.1% bovine serum albumin (BSA) for 30 min to reduce nonspecific binding. The scaffolds were then incubated with the primary antibody against CaSR (anti-surface calcium receptor, 1:100 dilution; Abcam, Cambridge, UK) prepared in 0.1% BSA at 4 °C overnight under gentle agitation.

**Figure 2:**
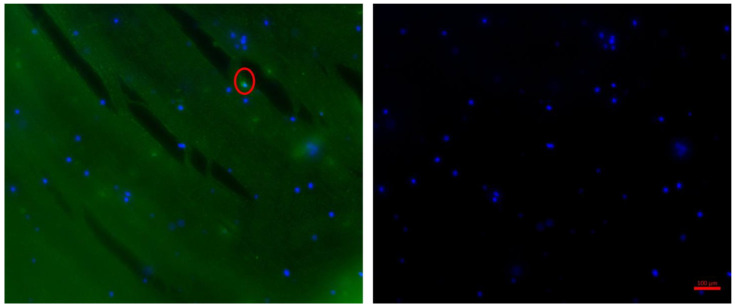
Anti-CaSR staining of isolated parathyroid cells within the 3D printed alginate scaffolds on day 1. The red circle indicates isolated and printed parathyroid cells.

After thorough PBS washes in order to remove unbound primary antibody, scaffolds were incubated with a fluorochrome-conjugated secondary antibody (Alexa Fluor^®^ 488-conjugated goat anti-mouse IgG, 1:200; Invitrogen, Waltham, MA, USA) for 1 h at room temperature in the dark. Nuclei were counterstained with 4′,6-diamidino-2-phenylindole (DAPI, 1 µg/mL in PBS) for 5 min. Finally, the stained scaffolds were mounted on glass slides using antifade mounting medium and imaged using a fluorescence microscope (Leica Microsystems, Wetzlar, Germany) (Figure 2).

### 2.4. In Vivo Implantation of 3D Scaffolds

A total of five 8-week-old male CD1 athymic nude mice (18–20 g) were used in this proof-of-concept in vivo study. Four mice received cell-laden 3D-bioprinted parathyroid scaffolds, with one animal allocated to each predefined post-implantation time point (1 week, 2 weeks, 3 weeks, and 3 months). Each mouse received a single scaffold implanted subcutaneously into the dorsal flank. In parallel, one mouse received an acellular alginate scaffold and served as the control. Animals were housed in individually ventilated cage (IVC) systems under controlled environmental conditions (23 ± 2 °C, 50% relative humidity, and filtered air) with free access to food and water. All experimental procedures were approved by the Hacettepe University Animal Experiments Ethical Committee (Approval No: 2022/646) and were performed in accordance with institutional and international guidelines for the care and use of laboratory animals.

**Figure 3:**
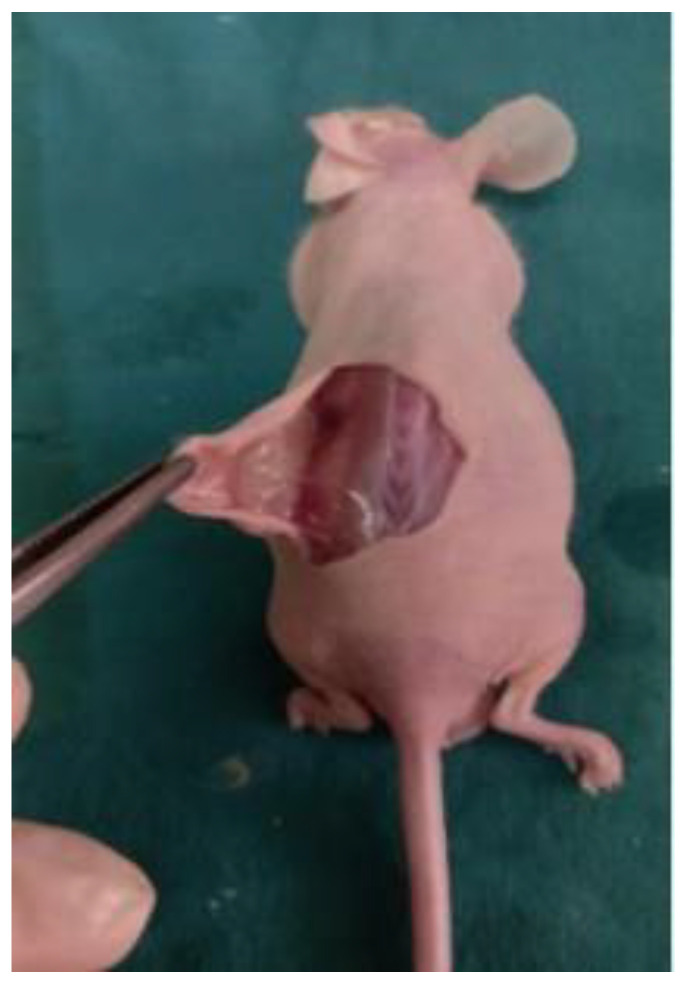
İmplanted 3D Bioprinted Scaffold.

For surgical implantation, anesthesia was induced with an intraperitoneal injection of ketamine (80–100 mg/kg) and xylazine (5–10 mg/kg). Parathyroid cell-laden 3D bioprinted scaffolds were implanted subcutaneously into the dorsal flanks of the mice to establish a parathyroid xenograft model. The surgical incision was closed using 5/0 monofilament absorbable sutures, and the animals were allowed to recover under standard IVC housing conditions (Figure 3).

At defined experimental time points (1 week, 2 weeks, 3 weeks, and 3 months post-implantation), scaffolds were carefully dissected from the mice, fixed in 10% neutral-buffered formalin, and processed for histological and immunohistochemical analyses. Parathyroid hormone (PTH) expression and secretion were specifically assessed by immunohistochemistry to evaluate the functional activity of the transplanted cells.

### 2.5. Histopathological Analysis

Bioprinted scaffolds together with the surrounding host tissue were excised at the indicated time points and processed for histological evaluation. Samples were fixed in 10% neutral-buffered formalin and subsequently dehydrated and infiltrated using an automated tissue processor. Paraffin-embedded blocks were prepared, and serial sections of 4–5 µm thickness were obtained using a microtome. Sections were mounted on glass slides and stained with conventional hematoxylin–eosin (H&E) according to standard protocols. Histological evaluation was performed under a light microscope to assess the general tissue architecture, cellular distribution, and the presence of viable parathyroid cells within the cell-laden scaffolds.

### 2.6. Immunohistochemistry

Paraffin-embedded tissue sections (4 μm thick) were prepared following standard fixation and dehydration procedures. Immunohistochemical staining for parathyroid hormone (PTH) was performed using a mouse anti-rat monoclonal PTH antibody (1:100; Cell Marque, Rocklin, CA, USA) on an automated staining platform (Ventana Benchmark XT, Roche, Tucson, AZ, USA). Antigen–antibody binding was visualized using the manufacturer’s recommended detection system, and sections were counterstained with hematoxylin. Stained slides were examined under a light microscope (Nikon Eclipse E600, Nikon Instruments Inc., Tokyo, Japan), and PTH expression was evaluated qualitatively to assess the functionality of the parathyroid cell–laden scaffolds.

## 3. Results

Human parathyroid cells were successfully isolated as freshly isolated primary human parathyroid cells and subsequently incorporated into 3D-bioprinted constructs. Within the alginate-based matrix, the cells exhibited a uniform morphological appearance and were evenly distributed throughout the scaffold (Figure 1A,B).

Immunofluorescence staining demonstrated that the bioprinted human parathyroid cells expressed the parathyroid-specific marker calcium-sensing receptor (CaSR) following isolation and bioprinting (Figure 2). CaSR expression was localized to the cell membrane surrounding the nuclei, confirming the preservation of parathyroid-specific phenotypic characteristics and functional identity within the 3D constructs.

**Figure 4:**
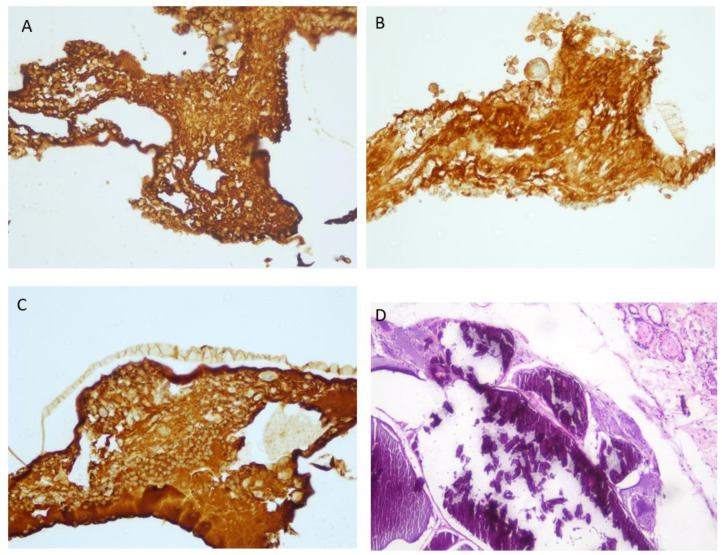
Paraffin embedded tissue sections of 4 μm thickness were processed with tissue fixation and dehydration. The Mouse anti-rat monoclonal PTH antibody (1:100; Cell Marque USA) staining on the BenchMark XT platform with Ventana’s detection systems. The expression of PTH was observed under a microscope (Nikon E600 ×400). ((**A**) At 1st week, (**B**) at 2nd week, (**C**) at 3rd week). (**D**) IHC was not deemed necessary because the parathyroid conjugates were found to be calcified, surrounded by foreign body tissue, and aphonic at the 3rd month (HE ×100).

**Figure 5:**
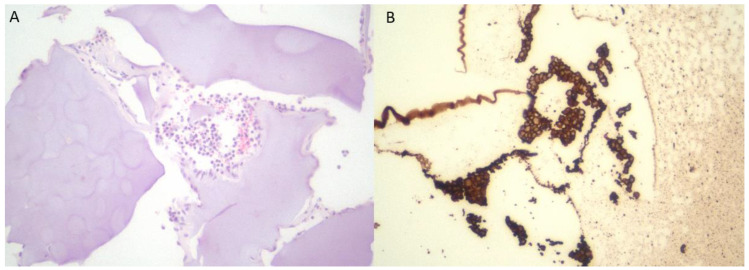
(**A**) Parathyroid cells and inflammatory cells in the pore of the scaffold were seen (HE ×200). (**B**) İmmunohistochemically parathyroid cells were positive for PTH (IHC ×200).

Cell-laden 3D-bioprinted scaffolds generated from primary human parathyroid cells were implanted subcutaneously into the dorsal flanks of nude mice under sterile conditions. The implanted constructs were explanted at predefined time points (1, 2, and 3 weeks post-implantation). With increasing implantation duration, a gradual increase in necrotic tissue was observed; however, the proportion of PTH-positive cells remained relatively stable during the first three weeks (Figure 4 and Figure 5).

Histological examination at later stages revealed that the scaffold pores were progressively filled with calcified deposits consistent with dystrophic calcification. The original scaffold architecture was largely replaced by dense fibrotic tissue. Within this fibrotic stroma, histiocytes and occasional foreign body-type multinucleated giant cells were observed adjacent to calcified areas, indicating a chronic foreign body reaction and loss of long-term graft viability.

No marked difference in PTH immunostaining intensity was observed among the first, second, and third weeks post-implantation, indicating preserved functional activity during the early period. In contrast, at the three-month time point, prominent calcification and fibrotic tissue replacement were evident within the scaffold, accompanied by loss of PTH expression. These findings demonstrate maintained short-term functionality but limited long-term graft viability.

## 4. Discussion

The most important result of this study was the characterization of human parathyroid tissue conjugates produced by the 3D bioprinting method in a xenograft athymic CD1 mouse model, using immunostaining and hormone secretion tests. The parathyroid conjugates dissected in the first, second, and third weeks were found to be alive and functioning. However, on the ninetieth day, the parathyroid conjugates were found to be calcified, surrounded by foreign body tissue, and aphonchiated.

When evaluating current surgical practices, the surgical techniques recommended for preventing hypoparathyroidism after thyroidectomy have not sufficiently reduced complication rates. The intraoperative identification of the parathyroid gland using Indocyanine Green (ICG) and the preservation of its vascularization is the most recent approach in the literature [23]. However, even with this technique, hypoparathyroidism rates have not been adequately reduced due to the rapid systemic distribution and short visualization time of ICG [24]. Data presented on predicting postoperative hypoparathyroidism may sometimes outweigh the support provided by intraoperative techniques. Dolidze et al. reported that the use of intraoperative parathyroid identification strategies, including 5-aminolevulinic acid–induced fluorescence and adjunct visual–instrumental techniques, significantly reduced the incidence of transient hypoparathyroidism and effectively prevented permanent hypocalcemia in thyroid surgery [25].

In thyroidectomies performed using robotic surgical techniques, the incidence of hypoparathyroidism remains comparable to that of conventional methods [26]. Hypoparathyroidism rates following bilateral axillo-breast approach robotic thyroidectomy and transoral robotic thyroidectomy range from 0.94% to 22% for transient cases, and 1.33% to 2.22% for permanent cases [27,28]. While some studies have shown a reduction in hypoparathyroidism rates with robotic techniques compared to conventional open thyroidectomy, it is still regarded as the most significant complication [29].

Although postoperative hypocalcemia is usually transient, it may become a severe and persistent complication when all parathyroid glands are inadvertently removed or devascularized, a scenario more commonly associated with total thyroidectomy rather than hemithyroidectomy. In routine surgical practice, autotransplantation of unintentionally removed or ischemic parathyroid glands is recommended. These glands, or those with suspected impaired blood supply, should be transplanted into the ipsilateral sternocleidomastoid or strap muscles [30]. A portion of the tissue intended for transplantation should be sent for frozen section analysis or subjected to rapid PTH testing to confirm it is parathyroid tissue [31]. If the vascular supply of the parathyroid gland is found to be compromised during surgery, or if parathyroidectomy has been performed, the affected gland should be considered the patient’s last functional parathyroid gland and should be autotransplanted [32].

Parathyroid transplantations are categorized into three types: parathyroid autotransplantation (PAT), parathyroid allotransplantation, and parathyroid xenotransplantation (PXT) [33]. Among these, autotransplantation, typically identified and performed during surgery, is the most common approach. The technique requires intraoperative recognition that the parathyroid gland has been excised, damaged, or has compromised vascularity. However, studies indicating that autotransplantation does not reduce the incidence of permanent hypoparathyroidism, and may even increase rates of transient hypoparathyroidism, have prompted the search for alternative solutions [34].

Cryopreservation of parathyroid tissue, followed by autotransplantation, is another option in cases of persistent surgical hypoparathyroidism in the postoperative period. For cryopreservation, the remaining tissue is sliced into small fragments, approximately 1 to 2 mm in size, and suspended in sterile saline before being frozen and stored at sub-zero temperatures in a tissue bank [32]. Re-implantation of cryopreserved parathyroid tissue is typically indicated within six months of surgery if the cervical remnants become nonfunctional [30,35]. Studies on delayed autotransplantation report success rates ranging from 17% to 83% [30,32,35,36].

An alternative treatment for patients under persistent HypoPT may be allotransplantation of cultured parathyroid cells combined with short-term immunosuppressive therapy [33]. Side effects associated with lifelong immunosuppression therapy are thought to have a negative impact on the outcome of this treatment. In the following years, many other case studies have been designed to solve the problem of histocompatibility and to reduce/prevent the need for immunosuppression treatment, and applications without immunosuppression have also been reported [36,37].

The classical treatment approach outlined in guidelines for post-surgical hypoparathyroidism has traditionally focused on calcium supplements and active vitamin D analogues [10,13]. However, with advancements in long-acting parathyroid hormone (PTH) analogues, alternative therapeutic options to conventional treatment have emerged. The introduction of PTH analogues, specifically PTH 1–34 and 1–84, represents a significant progression in managing hypoparathyroidism [38,39]. These analogues offer the potential to enhance quality of life by lessening patients’ dependency on frequent adjustments of calcium and vitamin D dosages. PTH analogue therapy facilitates serum calcium regulation and bone turnover normalization, thereby yielding substantial improvements in patients’ quality of life.

Long-acting PTH replacements further stabilize serum calcium levels, which helps to protect renal function and reduces hypercalciuria risk by minimizing calcium excretion. Nonetheless, rigorous monitoring of calcium balance and renal function remains essential, especially for those on prolonged PTH replacement therapy. Evidence from a phase 3 randomized controlled trial of TranCon PTH demonstrates these analogues’ efficacy in achieving eucalcemia, stabilizing bone mineral density, and normalizing bone turnover markers [39]. Despite these benefits, PTH-related analogues—already employed in osteoporosis treatment—present serious side effects, including potential malignancy and cardiovascular risks [40,41,42]. High-dose, long-term studies in rats indicate that all PTH and PTH-related protein (PTHrP) molecules pose a risk of osteosarcoma [41,43]. Consequently, they have yet to replace transplantation approaches that could replicate the natural parathyroid cycle, limiting their integration into routine clinical practice.

Generating functional endocrine organs using 3D bioprinting is relatively uncommon in the literature [21]. Most studies have focused on bioprinting tissues that are less vascularized, less innervated, and less functionally complex, such as cartilage and skin, rather than highly vascularized and functional organs like endocrine glands [44]). There are, however, some studies exploring parathyroid tissue engineering. For instance, Ritter et al. cultured parathyroid cells within a 3D collagen gel matrix [45], and another study achieved bioprinting of the thyroid gland using collagen hydrogel [46]. Additionally, biomaterials such as Matrigel, extracellular matrix, and alginate have been utilized in bioartificial endocrine gland models [44,46,47,48,49,50,51].

In our study, alginate was selected as the biomaterial to create a scaffold for parathyroid cells. Immunostaining experiments were employed to characterize the parathyroid scaffold. Literature findings indicate that surface calcium receptors (CaSR) are membrane-bound in functional parathyroid cells. As shown in Figure 2, anti-CaSR positive labeling was observed around the nucleus, confirming the successful development of a functional parathyroid scaffold in our study.

While vitamin D and calcium replacement therapies are standard in the traditional management of hypoparathyroidism, their limitations and practical challenges sustain the need for alternative treatments. Lifelong medication requirements and frequent blood tests present a particular burden, especially for younger patients. Additionally, insufficient replacement and persistent hypocalcemia pose heightened risks during pregnancy, advanced age, and for those with cardiac comorbidities, highlighting the need for calcium dose-dependent alternatives to current guideline-based treatments.

Although the exact mechanisms by which innate immunity contributes to transplant rejection are not fully understood, neutrophils and macrophages are believed to initiate the immune response following transplantation. Fibrotic rejection of implants may be linked to the early-stage activity of polymorphonuclear cells around the scaffold. In hypoparathyroidism management, parathyroid tissue constructs produced via calcium-sensitive 3D bioprinting have emerged as a promising physiological alternative, with the potential to address these limitations more effectively.

Recent advances in three-dimensional (3D) bioprinting have extended tissue engineering applications toward functionally active endocrine organs. Due to their small size, relatively simple microarchitecture, and hormone-driven function, endocrine glands represent particularly suitable targets for biofabrication [22]. Previous studies have demonstrated the feasibility of engineering hormone-active endocrine tissues, including thyroid, pancreatic islet, and adrenal constructs, primarily under in vitro conditions [46].

In this context, the present study provides novel in vivo evidence demonstrating short-term functional engraftment of 3D-bioprinted human parathyroid constructs in a xenograft model. Our findings reveal preserved PTH expression during the early post-implantation period, followed by time-dependent graft failure characterized by calcification and fibrosis. This temporal behavior underscores both the translational potential and current limitations of alginate-based scaffolds for parathyroid tissue engineering and highlights the importance of scaffold design, degradation kinetics, and host–graft interactions for sustained endocrine function in vivo.

This study has several limitations. Although multiple post-implantation time points were evaluated, the analyses were predominantly qualitative, and quantitative assessments of cell viability, PTH-positive cell proportions, and circulating or secreted PTH levels were not performed. Therefore, functional efficacy was inferred from marker expression rather than confirmed by direct hormone secretion assays.

In addition, the use of immunodeficient athymic mice limits the evaluation of long-term host–graft interactions and immune-mediated rejection. The fibrosis and calcification observed at later time points suggest limited long-term biocompatibility of the scaffold material.

Finally, this study was designed as a proof-of-concept for short-term functional engraftment of 3D-bioprinted human parathyroid constructs rather than to demonstrate clinical efficacy. Further studies incorporating quantitative functional analyses, optimized biomaterials, and immunomodulatory strategies are required for clinical translation.

## 5. Conclusions

The most significant result of this study is that, following the successful in vitro modeling of tissue constructs produced by our group using 3D bioprinting from human parathyroid tissue, in vivo modeling was also achieved in a xenograft model using athymic CD1 mice. Histopathological analysis confirmed that the tissue construction was functional during the first, second, and third weeks. However, by the third month, the implanted tissue had become calcified, was rejected due to a foreign body tissue reaction, and had lost its function.

The replication of a small portion of the parathyroid gland using 3D bioprinting for potential allotransplantation, following autotransplantation or cryopreservation in cases of intraoperative vascular compromise, remains a future project. The next critical phase of this research roadmap involves urgently studying the mechanisms of tissue rejection and evaluating the impact of immunosuppressants.

## Data Availability

Data are available upon reasonable request.

## Abbreviations

The following abbreviations are used in this manuscript:

3D	Three-dimensional
BSA	Bovine serum albumin
CaCl_2_	Calcium chloride
CaSR	Calcium-sensing receptor
CD1	Cluster of differentiation 1 (mouse strain designation)
CO_2_	Carbon dioxide
DAPI	4′,6-diamidino-2-phenylindole
DNA	Deoxyribonucleic acid
ECM	Extracellular matrix
FBS	Fetal bovine serum
FGF	Fibroblast growth factor
H&E	Hematoxylin and eosin
HypoPTH	Hypoparathyroidism
ICG	Indocyanine green
IF	Immunofluorescence
IHC	Immunohistochemistry
IL	Interleukin
IVC	Individually ventilated cage
MMPs	Matrix metalloproteinases
NK	Natural killer
NO	Nitric oxide
PBS	Phosphate-buffered saline
PAT	Parathyroid autotransplantation
PFA	Paraformaldehyde
PIGF	Placental growth factor
PMNs	Polymorphonuclear cells
PTH	Parathyroid hormone
PTHrP	Parathyroid hormone–related protein
PXT	Parathyroid xenotransplantation
RNA	Ribonucleic acid
ROS	Reactive oxygen species
RPMI	Roswell Park Memorial Institute medium
TGFβ1	Transforming growth factor beta 1
UV	Ultraviolet
VEGF	Vascular endothelial growth factor
